# A Century of Medical History in the County of Erie—1800–1900

**Published:** 1899-02

**Authors:** William Warren Potter

**Affiliations:** Buffalo, N. Y.


### A CENTURY OF MEDICAL HISTORY IN THE COUNTY OF ERIE.—1800–1900.

By WILLIAM WARREN POTTER, M. D., Buffalo, N. Y.

*Pioneer Physicians—Medical Societies—Medical Colleges—Hospitals—Medical Journals—Women Physicians—History of Homeopathy—Medical Officers of the Civil War—Individual Members of the Profession.*

[*Continued from the January edition.*]

### HISTORY OF HOMEOPATHIC MEDICINE.—*Continued.*[1]

THE Buffalo Homeopathic Hospital was organised June 14, 1872, and is located at 74 Cottage street, corner Maryland. We are unable to give the names of the first medical staff, but the board of trustees for the first year was made up as follows: Jerome Pierce, Charles C. McDonald, Benj. H. Austin, Sr., Loran L. Lewis, James Brayley, Francis H. Root, Jerome F. Fargo, John B. Griffin, Samuel V. Parsons, Mrs. C. C. Warner, Mrs. M. A. Kenyon, Mrs. Hannah Fargo, Mrs. Anna Poole Hoxsie, Mrs. Hattie E. Gregg,

1. This section was kindly furnished by Dr. F. Park Lewis.

Mrs. Charlotte E. Lewis. The capacity of the hospital is about sixty patients.

For a few years a rented building on Washington street was used, and then the site of the present hospital property was purchased and the building upon it remodeled for hospital purposes. Later an annex was added and an entire staff appointed, surgeons and other specialists having by this time found their way into the newer practice.

The present officers of the hospital are as follows:

Board of Trustees—President, George V. Forman; vice-president, M. H. Gratwick; secretary, Wm. Y. Warren; treasurer, Henry W. Burt; F. C. M. Lautz, Charles F. Dunbar, Henry W. Burt, John H. Meech, W. H. Gratwick, Jewett M. Richmond, George V. Forman, A. D. Gail, W. B. Miller.

Training School for Nurses—President, Dr. George R. Stearns; secretary, Mrs. Seth W. Warren; chairman, Mrs. David Sherrill.

President Board of Associate Managers, Mrs. C. J. North; superintendent of hospital, Mrs. Elizabeth Brainard; superintendent of nurses, Miss Josephine Snetsinger.

The present staff is constituted as follows:

Medical and surgical staff—President, Joseph T. Cook; first vice-president, Truman J. Martin; secretary, George T. Moseley. Consulting physicians—A. R. Wright, A. T. Bull, H. A. Foster, H. Baethig, A. M. Curtiss, John Miller, D. B. Stumpf. Attending physicians—E. P. Hussey, B. J. Maycock, E. A. Fisher, J. T. Cook, T. G. Martin, C. S. Albertson. Attending surgeons—general—H. C. Frost, D. G. Wilcox, G. T. Moseley; ophthalmic—F. Park Lewis; obstetricians, J. S. Halbert, G. R. Stearns; pathologist, A. W. Dods, Pharmacist, P. A. McCrea. Assisting physicians, A. B. Eadie, C. L. Mosher, W. D. Young. Assisting surgeons, M. F. Linquist, M. Manges, W. H. Marcy, H. L. Towner; ophthalmologists, W. A. M. Hadley, F. D. Lewis; obstetricians, Jessie Shepard, Rose Wilder; laryngologist, ————————. House staff—House physician, John G. Chadwick; house surgeon, C. E. Seaman.

A most important public work was undertaken in Erie county when the Collins Farm Hospital for the treatment of the insane was established by legislative enactment during the session of 1894. This is the second institution of this character under homeopathic management in the state. Extensive plans have been prepared for the buildings to be erected and $100,000 was appropriated during the last session of the legislature for this purpose. The first board of trustees consisted of William Tod Helmuth, M. D., New York city; Asa S. Couch, M. D., Fredonia; F. J. Blackman, Esq., Gowanda; Dr. Helmuth being elected president of the board.

In 1890, Dr. Dewitt G. Wilcox established a private hospital on the corner of Lexington and Elmwood avenues, which was known as the Wilcox Private Hospital. Subsequently a number of other physicians became interested in it, and it was incorporated as the Lexington Heights Hospital. It is largely devoted to surgical work, and is admirably equipped for this purpose.

The selection of a site for this institution, in a district which but for a few years previous had been stubble fields and open country, is not barren of suggestiveness. The growth of the young city in its physical geography was typical of a growth in other directions. There had gradually come into its mental atmosphere a new spirit. Men were not less earnest, less honest in their individual beliefs, or less tenacious of what they held to be truth, than heretofore. But a tolerance quite unknown to the previous generation began to be felt, a new respect for the opinions of others obtained among the best men of the two schools of practice.

This was the outgrowth, the natural consequence, of a wave of scientific thought which swept over the country, relieving the tension of puritanism in religion, modifying the conservatism of art—which had as yet no initiative life in America—loosening the bonds of tradition in literature, opening all that wide field of varied and beautiful writing which we now own proudly as distinctly American, and broadening and vivifying thought in every direction. It would be hard to say who laid the first spark to the brush heap of conventionalism. Many brave souls have tried to kindle it, but an enormous flaming torch was flashed from the old world to this when Charles Darwin sent across the water his doctrine of evolution. The effect of that first illuminating thought has not yet ceased—will never cease.

People began to realise that beliefs were never too old or too firmly set to be assailed. To some the whole fabric of life seemed menaced and covered with confusion. There may still be those who feel that the introduction of this revolutionary, investigative, scientific period into our history was an unmitigated evil. But its results were by no means merely iconoclastic. It opened men's minds clearly to a fact, too often forgotten, that truth cannot be carried in one small box.

That no one man or set of men ever had, or ever will have, all of truth, to the exclusion of all other men equally earnest in their search for the same truth, seems too axiomatic to need propounding. As a general statement it is accepted. Yet it has been the failure to realise this, in special instances, that has made possible the intolerant, aggressive spirit which, in one form or another, has been at the foundation of more than half of the wars and rebellions, insurrections and revolutions that this world has tragically witnessed.

It has been worth the price, then, of the mental suffering, doubt and even agnosticism, which a great upheaval like that following the introduction of such theories as that of evolution necessarily leaves, at least temporarily, in its train, to secure for the entire country a mental atmosphere less personal, more liberal, less dogmatic, more temperate, less concerned with individual feeling, more considerate of the aims and views of others, than that which formerly existed.

It may not be that Darwin's theory was itself responsible for these results, but it and the scientific thought of the time, following the lines laid down by Darwin, Huxley and others, called in question many long-established beliefs, clung to as fundamental by a large proportion of thinking people, and devotedly held, even to the point of martyrdom in days not so long in the past. This compelled people to think, and to think deeply; to think beneath and beyond preconceived opinion, to think in opposition to desire, in the face of despair; until, as in all such deep experience, out of this wrestling after truth came a sympathy and a consideration for others never before felt.

This, indeed, was recognised in all departments of life. Into art and literature, as well as into religious thought, it brought a spirit free, a temper reverent, and a method scientific. It was not to be wondered at, then, that the new school of medicine, which in its beginning had been hot-headed and radical, and the old school, which had been conservative and intolerant, should have renounced to a very large degree their bitterness and ill-feeling, and should be able to work together upon any occasion calling for their coöperation in the common duties of citizenship.

Such an occasion came in the spring of 1893, when the integrity of the city charter was threatened by political intrigues. This charter, which had been granted to the city by the state legislature, provided among other things a measure of local self-government, against which a political combination was directing its efforts. The citizens rose in revolt. The members of both county societies were called together in joint session to protest against the proposed legislation. Dr. P. W. Van Peyma was called to the chair, Dr. A. R.

Wright was made vice-president; Drs. Irving M. Snow and B. J. Maycock were chosen secretaries. Speeches were made by members of both societies and a committee of five, consisting of members from both county organisations, drew up resolutions upholding the city charter, and condemning any legislative action that would nullify it. Another joint committee was appointed by the chair to take any further action that might be deemed necessary in connection with other societies or clubs, the whole city being moved by the instinct of self-protection to protest against interference with its vested rights. The meeting then adjourned, this being the first meeting where physicians assembled upon a common platform without regard to school, since the separation which had occurred more than forty years before.

In the fall of 1893 another noteworthy event occurred. The infirmary department of the county almshouse had grown to very great proportions. A movement was successfully carried to take it out of the hands of a paid physician, and to place it under the charge of a large staff chosen from the leading physicians of the city. Upon this staff representatives of both methods of practice found place, and at the annual election, in evidence of the larger liberality and more generous feeling existing in the profession, the staff officers were chosen from both schools.

And thus, at the end of the century, the two schools of medicine, each of which has gone on increasing in strength and in power, although differing in some fundamental points of practice, toil side by side, working for a common cause. It may possibly happen that the future may so add to the science of the present, may so increase our knowledge of fundamental truth, that each may with full conviction and conscious dignity join fully with the other, in method as in hand, in the beneficent mission of the healing of the people. To-day we have at least reached the point of wishing each other "God speed."

(*Continued next month.*)

---

### ACETYLENE GAS-LIGHT FOR EXAMINATION OF THE EYES.

Appenzeller, of Rertlingen (*Centralbl. f. Prak. Augenheilkd.*, May, 1898), has a special apparatus with a 50-candle power burner which gives an absolute quiet, white light, particularly well adapted for ophthalmoscopic purposes. He considers his apparatus absolutely free from danger, and has used it many times a day with the greatest satisfaction. —*Canadian Practitioner and Review.*

